# Fast evolving 18S rRNA sequences from Solenogastres (Mollusca) resist standard PCR amplification and give new insights into mollusk substitution rate heterogeneity

**DOI:** 10.1186/1471-2148-10-70

**Published:** 2010-03-09

**Authors:** Achim Meyer, Christiane Todt, Nina T Mikkelsen, Bernhard Lieb

**Affiliations:** 1 Institute of Zoology, Johannes Gutenberg University, Müllerweg 6, 55099 Mainz, Germany; 2 Department of Biology, University of Bergen, Thormøhlens gate 53a, 5008 Bergen, Norway; 3 The Natural History Collections, Bergen Museum, University of Bergen, Muséplass 3, 5007 Bergen, Norway

## Abstract

**Background:**

The 18S rRNA gene is one of the most important molecular markers, used in diverse applications such as molecular phylogenetic analyses and biodiversity screening. The Mollusca is the second largest phylum within the animal kingdom and mollusks show an outstanding high diversity in body plans and ecological adaptations. Although an enormous amount of 18S data is available for higher mollusks, data on some early branching lineages are still limited. Despite of some partial success in obtaining these data from Solenogastres, by some regarded to be the most "basal" mollusks, this taxon still remained problematic due to contamination with food organisms and general amplification difficulties.

**Results:**

We report here the first authentic 18S genes of three Solenogastres species (Mollusca), each possessing a unique sequence composition with regions conspicuously rich in guanine and cytosine. For these GC-rich regions we calculated strong secondary structures. The observed high intra-molecular forces hamper standard amplification and appear to increase formation of chimerical sequences caused by contaminating foreign DNAs from potential prey organisms. In our analyses, contamination was avoided by using RNA as a template. Indication for contamination of previously published Solenogastres sequences is presented. Detailed phylogenetic analyses were conducted using RNA specific models that account for compensatory substitutions in stem regions.

**Conclusions:**

The extreme morphological diversity of mollusks is mirrored in the molecular 18S data and shows elevated substitution rates mainly in three higher taxa: true limpets (Patellogastropoda), Cephalopoda and Solenogastres. Our phylogenetic tree based on 123 species, including representatives of all mollusk classes, shows limited resolution at the class level but illustrates the pitfalls of artificial groupings formed due to shared biased sequence composition.

## Background

The small subunit (SSU) 18S rRNA gene is one of the most frequently used genes in phylogenetic studies (see below) and an important marker for random target PCR in environmental biodiversity screening [1]. In general, rRNA gene sequences are easy to access due to highly conserved flanking regions allowing for the use of universal primers [2]. Their repetitive arrangement within the genome provides excessive amounts of template DNA for PCR, even in smallest organisms. The 18S gene is part of the ribosomal functional core and is exposed to similar selective forces in all living beings [3]. Thus, when the first large-scale phylogenetic studies based on 18S sequences were published - first and foremost Field et al.'s [4] phylogeny of the animal kingdom - the gene was celebrated as the prime candidate for reconstructing the metazoan tree of life. And in fact, 18S sequences later provided evidence for the splitting of Ecdysozoa and Lophotrochozoa [[5], see also [6]], thus contributing to the most recent revolutionary change in our understanding of metazoan relationships. Methodological innovation within the last years came from the incorporation of secondary structure into phylogenetic analyses. In particular RNA specific substitution models considering paired sites in rRNA genes have been shown to outperform standard DNA models [7-12].

During recent years and with increased numbers of taxa included into molecular phylogenies, however, two problems became apparent. First, there are prevailing sequencing impediments in representatives of certain taxa, such as the mollusk classes Solenogastres and Tryblidia [13,14], selected bivalve taxa (pers. comment H. Dreyer), and the enigmatic crustacean class Remipedia (pers. comment H. Glenner). Failure to obtain 18S sequences of single taxa is considered a common phenomenon but is rarely ever reported. Secondly, in contrast to initially high hopes, 18S cannot resolve nodes at all taxonomic levels and its efficacy varies considerably among clades. This has been discussed as an effect of rapid ancient radiation within short periods [15]. Multigene analyses are currently thought to give more reliable results for tracing deep branching events in Metazoa but 18S still is extensively used in phylogenetic analyses.

Considering the wide range of studies based on the 18S gene as a molecular marker, both sequencing problems and the applicability of 18S for phylogenetic inferences need to be scrutinized. To address these questions, we focus on the Mollusca, the second largest animal phylum. There are eight higher taxa (classes) defined within Mollusca: the aplacophoran Solenogastres (= Neomeniomorpha) and Caudofoveata (= Chaetodermomorpha), two small clades with about 250 and 150 currently described species; Polyplacophora (ca. 920 species); and the conchiferan clades Tryblidia (= Monoplacophora; 29 species described), Scaphopoda (ca. 520 species), Cephalopoda (ca. 1,000 species), Bivalvia (ca. 30,000 species), and Gastropoda (40,000-150,000 species). The monophyly of the phylum is well established based on morphological characters, but 18S phylogenies often show the Mollusca as polyphyletic or paraphyletic with low resolution of the deeper nodes [e.g: [16-19]]. One of the main deficiencies of all published studies on mollusk phylogeny is the underrepresentation of the minor taxa Solenogastres, Caudofoveata, and Monoplacophora. The aplacophoran Solenogastres and Caudofoveata together with Polyplacophora (chitons) are traditionally thought to represent early branching taxa within Mollusca, but their relative position is still controversially discussed [for review see [20,21]]. Despite of their key position in mollusk phylogeny and evolution, the number of molecular phylogenetic studies including any aplacophoran mollusk species is low: Winnipeninckx et al. [22]; Okusu et al. [14]; Lindgren et al. [23]; Passamaneck et al [19]; Giribet et al. [13]; Dunn et al. [24]; Wägele et al. [25]; Wilson et al. [26]. To date there are no more than four caudofoveate and five solenogaster (partial) 18S sequences published in Genbank, the taxon sampling spanning no more than two genera of Caudofoveata and Solenogastres, respectively.

In solenogasters, Okusu and Giribet [14] described severe contamination issues caused by cnidarian prey. Here, we specifically address contamination issues and point out technical problems hampering amplification during PCR. This is important not only for prompting representative taxonomic sampling for phylogenetic analyses, but also for avoiding under- or overestimation of biodiversity in environmental screening programs. Moreover, we evaluate the usefulness of the 18S gene for phylogenetic inferences by combining our new authentic solenogaster 18S data with published molluscan sequences and - based on an extended taxon sampling - analyze sequence divergence and compositional heterogeneity within the phylum.

## Results

### Solenogastres sequences

Initial experiments using standard PCR protocols and genomic DNA from starved specimens resulted in sequences from prey organisms and epibionts, or in chimerical PCR products. At best, cDNA templates led to shortened 18S sequences. Finally, authentic solenogaster 18S sequences for three species were obtained via isolation of total RNA from starved specimens, followed by reverse transcription and utilizing additives for GC-rich templates. 10% DMSO was applied in sequencing reactions.

**Figure 1:**
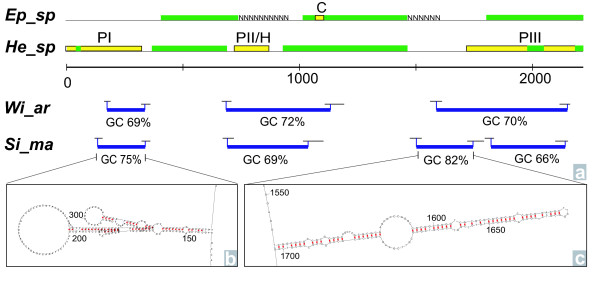
**Helical regions correspond with missing data or possible foreign DNA**. **1a**: Possible chimerical patterns in previously published sequences (yellow bars) match with regions of double stranded DNA forming helical regions (blue bars) in newly generated sequences. *Epimenia *sp. (Ep_sp) and *Helicoradomenia *sp. (He_sp) sequences have been aligned to the nucleotide positions (represented by the scale bar) of the sequences from this study, *Wirenia argentea *(Wi_ar) and *Simrothiella margaritacea *(Si_ma). The simplified schematic alignments (green bars) show high similarity (>90%) whereas other regions (yellow bars) possess lower similarity and point to contamination issues. BLASTn searches of yellow domains indicate high similarity with polychaetes (PI to III) or cnidarians (C), or resulted in no significant hit, indicated as *Helicoradomenia *only (H). Detailed BLASTn results are given in additional file 1: Blast result table. Below the scale bar, the double stranded helical regions of 18S sequences are indicated containing 66-82% GC islands. **1b **and **c**: Close-up views of single stranded regions of helix 1 (1b) and helix 2 (1c) of *S. margaritacea *18S. These stems have strong adhesive forces probably hampering PCR. Secondary structures were calculated using mfold, applying 72°C. G-C hydrogen bonds are indicated in red.

Strong secondary structures and GC clamps were observed analyzing the *Wirenia argentea *(2161 bp, GC content = 63.12%), *Simrothiella margaritacea *(2149 bp, GC content = 61.42%), and *Micromenia fodiens *(2087 bp, GC content = 62.72%) 18S sequences. Stitch Profiles [27] computes the temperature-dependent melting probability and location of dsDNA (helical) and ssDNA regions and thus can be used to locate the formation of DNA bubbles at a predefined temperature. We determined three to four helical sections in solenogaster dsDNA (figure 1a, blue bars). These regions additionally bear prominent stems in ssDNA, as inferred with the secondary structure probability plot in MFold at 72°C (Figure 1b, c). Elevated GC contents above 60% were observed in the helical regions, with a maximum of 82% in the second helix of *S. margaritacea*.

**Figure 2:**
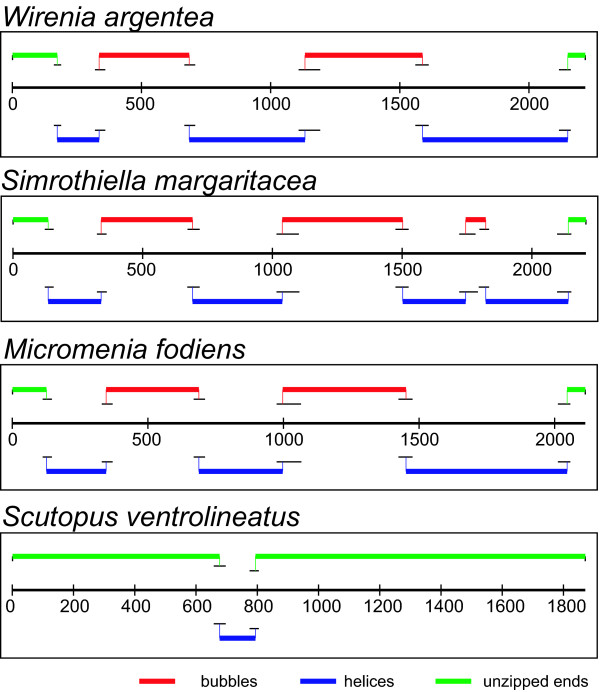
**Secondary structures of double stranded 18S sequences**. Stitch Profiles calculated at 90.7°C showing melted and double stranded regions for the three new solenogaster 18S sequences and the caudofoveate *Scutopus ventrolineatus*. One short helical region in the *S. ventrolineatus *sequence of approximately 100 bp length and 63% GC content is at the same position as in the solenogaster sequences.

A previously published complete caudofoveate (*Scutopus ventrolineatus*) 18S sequence showed only one short helical region (~100 bp, GC content 63%), which could be aligned to the second helix of our solenogaster sequences (figure 2). All previously published solenogaster 18S sequences lack exceptional secondary structures and the GC contents are below 60%. The *Epimenia *sp. sequence includes a 46 bp fragment with 100% cnidarian sequence identity (figure 1; yellow bar, C). The two nearly complete *Helicoradomenia *sp. sequences show 79.3% identity to each other. Analyses of selected sections from these sequences using BLASTn searches showed significant identities of >95% with the polychaete *Amphisamytha galapagensis *and other polychaetes (See additional file 1: Blast result table). Sections with missing data or exogenous DNA in previously published sequences match the regions of strong secondary structures determined within the sequences of *W. argentea, S. margaritacea *and *M. fodiens *(figure 1, yellow bars: PI, PII/H and PIII and figure 2).

### Sequence composition and alignment

Among the 829 mollusk taxa sampled for 18S, the Cephalopoda, Solenogastres and Patellogastropoda (table 1) show a combination of high GC content (>57%) and elongated gene length (>2,000 bp). The variability within the selected taxa is measured with the disparity index (*I*_*D*_), which has been shown to be more powerful than the commonly used χ^2 ^test to assess substitution patterns [28]. The compositional heterogeneity varies within the selected mollusk taxa and is not necessarily correlated with the sample size. For example, the disparity index within the 116 Caenogastropoda (*I*_*D *_= 0.01) is remarkably low whereas Heterobranchia is highly variable (*I*_*D *_= 1.86). The estimation of average Maximum Likelihood (ML) branch lengths required the recoding of 4,315 ambiguously aligned and highly variable positions as binary presence/absence data from 5,918 overall positions. The highest-ranking ML branch length values are found for Cephalopoda, Solenogastres and Patellogastropoda (table 1, column 7).

**Table 1:** Sequence composition within selected taxa (no = number of specimens) characterized using the disparity index (*I*_*D*_), compositional distance (Dc), percent GC content, gene length in base pairs (bp) and average branchlengths (brls) with standard deviation (σ).

Taxa	No	***I***_***D***_	Dc	GC	bp	Brls*10	σ*10
Outgroup	41	0.42	0.52	48.5	1682.9	0.60	0.41
**Polyplacophora**	32	0.08	0.11	49.9	1739.8	0.46	0.08
**Monoplacophora**	1	n/c	n/c	50.4	1767.0	0.38	n/c
**Scaphopoda**	22	0.14	0.21	53.5	1835.8	1.47	0.19
**Caudofoveata**	3	0.00	0.01	55.8	1785.0	1.82	0.08
**Solenogastres**	3	0.18	0.23	63.9	2100.0	5.27	0.24
**Cephalopoda**							
Coleoidea	74	0.19	0.28	60.3	2275.3	7.48	1.37
Nautiloidea	3	0.31	0.34	52.9	2360.0	5.40	0.35
**Gastropoda**							
Vetigastropoda	31	0.03	0.08	51.2	1784.9	2.10	0.12
Neritimorpha	5	0.00	0.02	50.6	1736.0	0.43	0.05
Neomphalida	3	0.09	0.14	51.3	1790.7	1.48	0.02
Cocculinida	1	n/c	n/c	50.1	1749.0	4.11	n/c
Patellogastropoda	9	0.42	0.50	57.2	2011.9	5.99	0.05
Caenogastropda	116	0.01	0.02	50.7	1745.7	0.86	0.05
Heterobranchia	183	1.86	1.96	55.3	1796.5	2.18	1.54
**Bivalvia**							
Protobranchia	18	0.12	0.17	50.6	1751.6	0.46	0.24
Paleoheterodonta	13	0.02	0.05	49.6	1750.5	0.47	0.08
Pteriomorpha	111	0.06	0.10	49.6	1742.1	0.48	0.38
Heterodonta (ex. Anom.)	160	0.25	0.34	52.2	1760.2	2.01	0.48
Anomalodesmata	41	0.11	0.20	51.7	1874.7	2.86	0.27

* Brls and σ values were multiplied with 10 for better readability. We used all sequences available for the Mollusca, Sipuncula and Kamptozoa with a length over 1,600 bp, supplemented by some other Lophotrochozoa and *Priapulus caudatus*.

Sequence composition of Mollusca within selected taxa measured using the disparity index (*I*_*D*_), compositional distance (CD), GC content [%], gene length in base pairs (bp) and average branch lengths (brls) and their standard deviation (σ). Brls and σ values were multiplied with 10 for better readability. We used all sequences available for the Mollusca, Sipuncula and Kamptozoa above 1,600 bp supplemented by several other Lophotrochozoa and *Priapulus caudatus*.

This initially analyzed large dataset also guided the taxon selection for the phylogenetic analyses. The resulting 123 taxa prank alignment comprises 9,027 aligned positions and 2,051 positions past trimming. The proportion of gaps or completely undetermined characters (N) in the final alignment is 17.5%. The average disparity indices between the groups of sequences from this alignment show Solenogastres distinct from all other mollusks except cephalopods (*I*_*D *_< 1) and are depicted in the lower triangle of table 2. Additionally, we tested the homogeneity of substitution patterns calculating all pair-wise sequence comparisons in Mega 4.0 [29]. Significance with α≤0.01 was tested with 1,000 Monte Carlo replicates using the pair-wise deletion option. The substitution pattern of the three Solenogastres sequences from this study are significantly different from all taxa except for Cephalopoda, most Patellogastropoda and some heterobranchs (23 out of 30 pair-wise comparisons rejected homogeneity with α≤0.01: 90%; see table 2, upper triangle).

**Table 2:** Results from the disparity analyses on the 123 taxon alignment with the disparity index between taxa depicted in the lower triangle and the mcmc-test results for the homogeneity of substitution pattern in the upper triangle (% of taxa).

Taxa (No)	Anom	Caeno	Caudo	Ceph	Cocc	Hetb	Mono	Neom	Nerit	Paleo	Pate	Polyp	Protob	Pterio	Scaph	Solen	Hetd	Vetig
Anomalodesmata (7)		0	100	85	0	39	0	0	0	5	90	0	0	0	46	**100**	37	0
Caenogastropoda (7)	0.029		100	98	0	56	0	0	0	0	100	0	21	0	83	**100**	55	0
Caudofoveata (2)	1.303	1.626		19	100	50	100	100	100	100	17	100	100	100	50	**100**	32	100
Cephalopoda (16)	3.350	4.198	0.764		81	85	88	85	90	94	29	95	85	91	29	31	78	90
Cocculinida (1)	0.004	0.000	2.029	3.793		40	0	0	0	0	100	0	0	0	40	**100**	36	0
Heterobranchia (10)	0.898	1.189	0.719	2.153	1.155		50	43	58	73	43	70	57	62	54	90	47	44
Monoplacophora (1)	0.003	0.001	1.685	4.100	0.000	1.083		0	0	0	100	0	22	0	80	**100**	64	0
Neomphalida (3)	0.006	0.121	1.122	3.182	0.042	0.886	0.045		0	56	78	52	11	17	27	**100**	21	0
Neritimorpha (5)	0.008	0.001	1.767	4.451	0.000	1.228	0.000	0.095		7	93	0	24	3	88	**100**	64	0
Paleoheterodonta (3)	0.075	0.043	2.412	5.173	0.007	1.629	0.054	0.270	0.047		100	0	33	0	100	**100**	81	0
Patellogastropoda (3)	2.152	2.488	0.581	0.586	2.551	1.041	2.382	1.808	2.245	3.077		100	85	100	40	44	69	100
Polyplacophora (7)	0.114	0.015	2.430	5.259	0.007	1.579	0.030	0.311	0.035	0.025	2.974		30	0	100	**100**	84	0
Protobranchia (9)	0.049	0.138	1.419	3.690	0.091	1.035	0.102	0.088	0.129	0.252	1.972	0.275		28	53	**100**	63	2
Pteriomorpha (6)	0.056	0.014	2.112	4.483	0.000	1.383	0.028	0.200	0.021	0.010	2.560	0.020	0.207		90	**100**	80	0
Scaphopoda (5)	0.397	0.727	0.478	1.869	0.546	0.590	0.625	0.260	0.734	1.064	0.755	1.123	0.489	0.916		**100**	19	42
**Solenogastres (3)**	**5.316**	**6.006**	**1.408**	**0.944**	**6.180**	**3.215**	**5.999**	**4.886**	**6.364**	**7.748**	**1.046**	**7.407**	**5.487**	**6.788**	**3.291**		**100**	**100**
Heterodonta s.A. (14)	0.339	0.503	0.507	2.314	0.532	0.673	0.487	0.224	0.520	0.906	1.365	0.882	0.430	0.741	0.208	**3.456**		56
Vetigastropoda (9)	0.022	0.048	1.219	3.578	0.039	0.970	0.026	0.030	0.039	0.144	2.195	0.131	0.098	0.083	0.376	**5.174**	0.438	

* We considered homogeneity as significantly rejected with P- values ≤ 0.01. The pairwise deletion option was used to compare all possible species pairs (100% = all pairwise comparisons between the species from both taxa rejected homogeneity). Anomalodesmata were treated separately and thus are excluded when showing results for the remaining Heterodonta.

### Phylogenetic analyses

**Figure 3:**
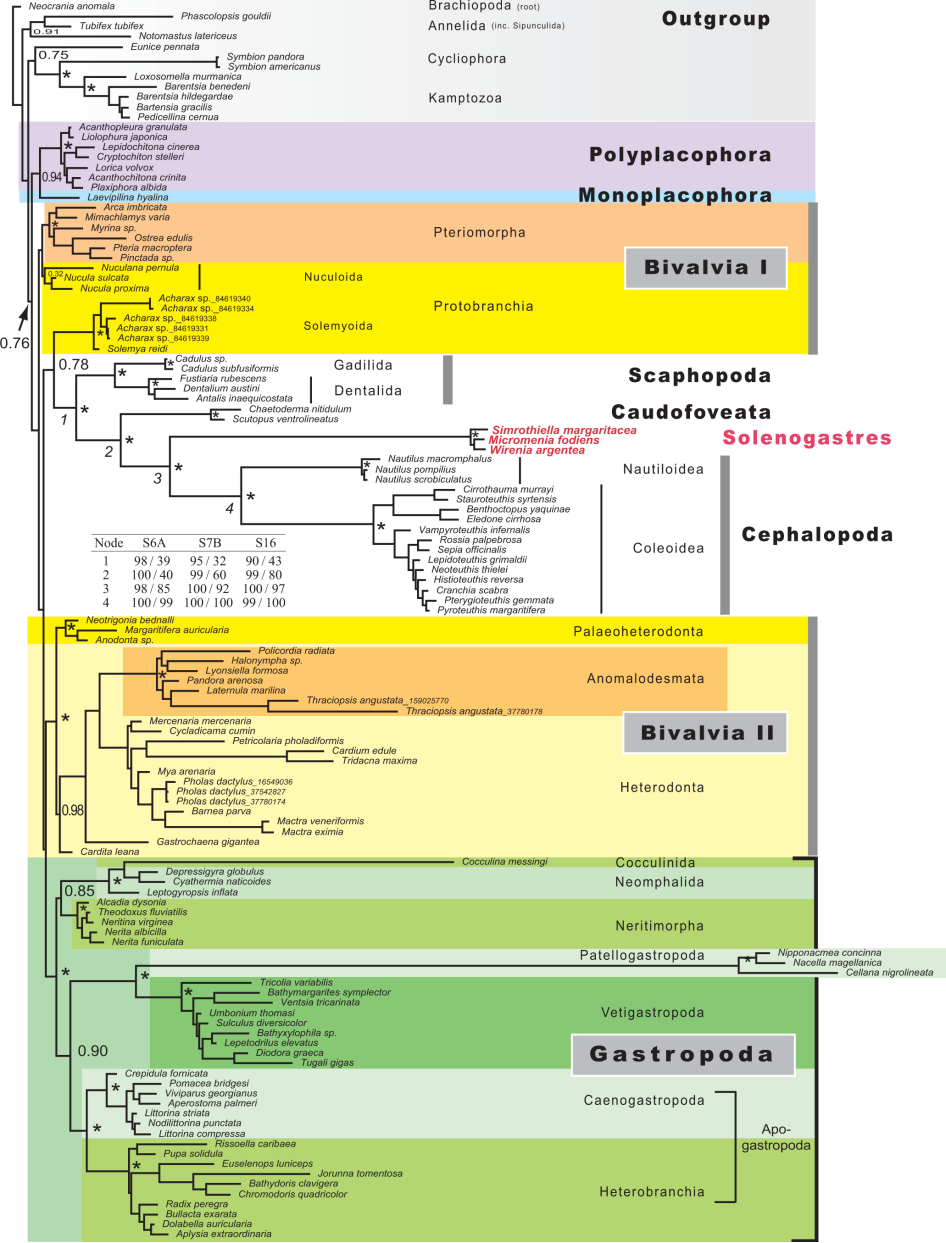
**Consensus tree from the bayesian analysis using a time heterogeneous model in phase 2.0**. * = indicates full support from the posterior probability (pp: 1.00). The Mollusca (pp: 0.75) and all mollusk classes (pp: 1.00) except Bivalvia were recovered. Solenogastres and Cephalopoda were assigned to different substitution models within this run. The branching pattern assembling taxa with similar sequence composition at node one to four is very robust in all analyses (see also table 2). Inserted table: ML bootstrap support (BS) is given for the nodes 1-4 using three suggested doublet models (S6A, S7B, S16). We used two different coding schemes for unpaired sites to infer the possible influence of base composition to the results. BS values are depicted in the order NT/RY: original nucleotide (NT) coded loops in followed by the results providing a recoded (G, A: R; T, C: Y) loop region.

**Figure 4:**
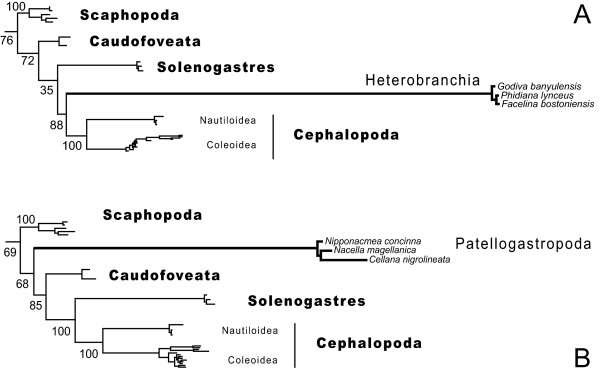
**ML analyses with a modified taxon sampling**. **A**: The heterobranchs are here represented by representatives of the fast evolving Aeolidina, only. All other taxa are identical to the sampling shown in figure 3, but we excluded also Patellogastropoda. **B**: Same alignment as used in the inference for figure 3 but excluding all other gastropods except Patellogastropoda. Both analyses were conducted on ten different starting trees with a partitioned GTR + and S16 substitution model and enabling the bootstopping criterium in RAxML (-# autoFC).

Only the analysis using Phase 2.0 [30] with the time heterogeneous model found the Mollusca monophyletic, albeit with low support (arrow, figure 3). We found full support for all class level taxa except Bivalvia and confirmed all the well-established subgroups within the Gastropoda, Cephalopoda, and Scaphopoda. Polyplacophora and the single monoplacophoran representative together form the first branch of the molluskan tree. Within bivalves, Palaeoheterodonta and Heterodonta form a robustly supported clade. Pteriomorpha and Protobranchia are outside this clade. Protobranchia appears paraphyletic, with Nuculoidea representing the sister group to Pteriomorpha and with Solemyoida included into a clade comprising Scaphopoda, the aplacophoran clades, and Cephalopoda. Both Bayesian and ML analyses revealed an identical internal branching pattern of (((Solenogastres+Cephalopoda)+Caudofoveata)+Scaphopoda). The calculated ML bootstrap support values for this topology using different models and coding schemes are tabulated in figure 3. All conducted analyses agree with large parts of the tree. The ML topologies corresponding to the three best fitting RNA doublet models offer only minor topological differences as judged by eye and using the weighted Robinson Foulds distance (wRF) [31] accounting for node support values (average relative wRF: 0.027931). RNA doublet models of different complexity are not nested, thus we only determined the best fitting model within each class of substitution models using the Akaike Information Criterion (AIC) [32]. The number of states describes the treatment of the possible ten non-Watson-Crick pairs, which account all together for 16% miss-matches in the 123 taxa alignment. Each model differs in the number of free parameters (k). The favored six state model is S6A (k: 19+9, AIC: 76557.9287). Within the seven state models S7B (k: 23+9, AIC: 83726.0469) and within the 16 state models S16 (k: 134+9, AIC: 87394.3873) were selected. We choose the S7B substitution model as a trade off between computational demand and complexity in the Bayesian analyses. The Phase software is designed to infer phylogenies from rRNA genes with paired sites and is able to model compositional heterogeneity [30]. The likelihood traces and most of the model parameters entered the stationary phase between 500,000 and 1,000,000 generations (Additional file 2). All five chains using the time heterogeneous model resulted in the identical branching pattern of the respective consensus tree and only minor differences in branch lengths. The consensus tree was calculated from a chain sampling 50 mio generations to maximize the effective sample size (ESS) for all estimated parameters. From a total of 120 model parameters, six failed to extravagate the ESS of 200, and four parameters were only sparsely sampled (ESS < 100). To further investigate the possible influence of exceptional sequence composition of Solenogastres (see table 2) we modified the taxon sampling of gastropods in two analyses. (i) All Patellogastropoda and Heterobranchia in the alignment were excluded and replaced by three fast evolving Aeolidina (Heterobranchia). (ii) All gastropods were excluded but Patellogastropoda. Both experiments resulted in a regrouping of the above mentioned (((Solenogastres+Cephalopoda)+Caudofoveata)+Scaphopoda) clade placing Patellogastropoda between Scaphopoda and Caudofoveata or proposing Aeolidina as sistergroup to Solenogastres and Cephalopoda (figure 4). All trees and alignments are available from the authors.

## Discussion

### GC rich 18S and the chimera problem

Amplification problems are a frequent phenomenon, even if using standard markers such as the 18S rRNA gene. Secondary structures and GC-rich sections as pronounced as for the solenogaster 18S RNAs shown here, have not been reported earlier. May GC-rich sequences cause hampered PCR reactions in other taxa, too? Based on our results, we assume this to be likely, but equally startling as the failure to obtain gene sequences is the danger to produce chimerical products.

GC-rich sequences demand higher melting temperatures to be converted into and kept as single stranded DNA molecules. Under standard amplification conditions, sequence sections exclusively composed of GC residues will terminate elongation steps in PCR by forming highly stable stems or GC clamps causing amplification to be refractory. Incompletely extended primers can anneal to heterologous 18S sequences and, despite of some degree of nucleotide mismatching, they will often be completed in the subsequent polymerization step resulting in chimeras [33]. Alternatively, compatible priming sequences from genes with lower GC contents are favored and successfully amplified [34]. Single stranded sequences with pronounced GC-stem regions also may lead the Taq polymerase to skip the 'locked' sections, which leads to shortened PCR products lacking the stem-loop regions [e.g.: [35]]. This potentially explains the lack of data for the GC-rich regions in the previously published solenogaster sequences, in contrast to the *Wirenia argentea*, *Simrothiella margaritacea*, and *Micromenia fodiens *sequences described herein. This interpretation is corroborated by the severe amplification problems we experienced when using recombinant plasmids as templates under standard PCR conditions (denaturing step at 94°C). The cloned *Wirenia argentea *18S inserts could not be amplified using conventional PCR protocols but resulted in blank agarose gels (not documented), although the number of templates accessible from clone amplification usually exceeds the gene copy number of genomic DNA extractions by far. Failure to amplify solenogaster 18S under such conditions demonstrates that clean starting material is just the first step. In addition to ours, a number of protocols for difficult or GC rich templates are published [36,37], supplemented by several commercial kits.

### Contamination

The solenogaster midgut is extremely voluminous and can hold undigested food that may provide considerable amounts of DNA templates leading to non-target or chimerical amplification products (see above). To avoid contamination with prey organisms, Okusu and Giribet [14] suggested the use of gonad tissue, larval material or of species that are not predators of metazoan organisms. The first two options are feasible where material is available, but the third option can be misleading when the diet is unknown. A prey-predator relationship between *Helicoradomenia *and non-cnidarian metazoan organisms, probably polychaetes, has been proposed earlier based on transmission electron microscopy data [38].

Starving animals in the laboratory for several days can reduce the amount of contaminating prey tissue considerably [39], but exogenous DNA was amplified even after starvation times of up to three months when using standard PCR protocols. This may be due to residues of prey cnidocysts held back in the midgut epithelial cells. Thus, we found isolation of total RNA from starved specimens, followed by reverse transcription, to be most effective to avoid contamination. Isolation of total RNA followed by DNase digestion makes up to 95% of rRNA available for RT-PCR and destroys contaminating non-target DNA templates. Due to the ubiquitous presence of RNases causing instability of RNA outside of living cells, the presence of considerable amounts of exogenous RNA within an isolate is highly unusual, but one always has to bear in mind the possibility of contamination with parasites, epibionts, and undigested prey tissue.

### Phylogenetic inferences

Considering the pitfalls of phylogenetic analyses based on a single gene and taking into account well-established knowledge on mollusk relationships, a number of groupings in our tree are ambiguous. This concerns for example the clade composed of Cephalopoda and Solenogastres, where molecular inferences may reflect a shared bias in base composition rather than a sistergroup relationship. This assumption is corroborated by the two experiments testing the influence of taxon sampling by manipulating the gastropod dataset (figure 4). In Patellogastropoda and Heterobranchia there are species with substitution patterns similar to Solenogastres (table 2). For both taxa, the exclusion of species breaking up their long branches resulted in a placement next to species with a similar sequence composition. The branching pattern including all major gastropod lineages is robust in all analyses both within the Gastropoda and the clade (((Solenogastres+Cephalopoda)+Caudofoveata)+Scaphopoda). When we add the heterobranch taxa from the original analysis to the three extremely fast evolving Aeolidina, the latter taxon is correctly placed inside the heterobranch radiation (data not shown). Within Solenogastres our species selection aimed to reflect the taxon diversity by selecting representatives from two different orders (three families), but the trimming of the alignment adapted to the broad taxon sampling diminished the phylogenetic signal at the species level.

Our new and more representative dataset of 123 taxa covered for the first time all mollusk classes, but our analyses across the Mollusca resulted in short internal branches combined with single long branching clades. This scenario is known to be critical in phylogenetic tree reconstructions [40]. The structure aware and non-stationary models greatly improved the results by detecting "problematic" taxa and by resulting in increased node support for well established subgroups. Thus we were able to recover nearly all mollusk classes, even though problematic fast evolving taxa, such as Patellogastropoda, were included. In rejecting the major mollusk clade Conchifera (mollusks with a single or bivalved shell), however, our phylogenetic tree does not reflect traditional views. We detected a sister group of Polyplacophora and Monoplacophora as proposed by Giribet et al. [13] and Wilson et al. [26], but not with full support. Both taxa show only minor differences in sequence composition (table 2), which might mislead analyses of class level relationships.

The failure to detect monophyletic Bivalvia probably best demonstrates the limits of 18S analyses in Mollusca using contemporary methods [see also [19,22,41]]. Bivalvia are known to show a number of exceptional morphological characters as well as several unusual structural features in the mitochondrial genome [42]. Thus, despite not obviously fast evolving, here the alignment methods may have failed to identify homologous positions correctly. Alternatively, the phylogenetic signal in bivalves may be largely eroded. The close relationship between Scaphopoda and Protobranchia is worth mentioning because it (partly) supports the so-called Diasoma hypothesis, recently rejected by a number of molecular and morphological analyses, but here again proposing a sistergroup relationship between Bivalvia and Scaphopoda [43],

Across all analyses we recovered a previously proposed clade composed of Cycliophora and Kamptozoa within the outgroup [44-47].

### Sequence divergence in Mollusca

Elevated substitution rates are known to be gene specific but also characteristic for certain lineages across different genes [48,49]. High substitution rates in the generally well-conserved 18S gene thus may point to fast evolving taxa. To allow for general conclusions across the Mollusca, we inferred substitution rates for all published molluscan 18S sequences longer than 1600 bp. The solenogaster 18S sequences obtained in this study are among the fastest evolving 18S sequences within the Mollusca. Solenogastres bear a number of assumed ancestral mollusk features, but substitution rates in the 18S gene are nearly doubled in our selected species compared to other presumably "basal" mollusks, such as Caudofoveata and Polyplacophora. If the assumed plesiomorphic morphological characters in Solenogastres are in fact conserved ancestral features, then molecular and morphological rates of evolution are unlinked, at least for the 18S gene. Similar cases of possibly ancestral morphology combined with exceptionally high substitution rates as shown in table 1 are *Nautilus*, the "living fossil" cephalopods, and Patellogastropoda, the true limpets [50,51]. A number of factors, for example functionality of proteins and RNAs, generation time, metabolic rate, population size, and life histories, are thought to influence substitution rates [52,53].

## Conclusions

We show that the solenogaster 18S gene has an exceptional base composition, resulting in a number of technical difficulties. Amplification problems are a common phenomenon but can be overcome by combining known methods in a new framework and by employing alternative strategies. We suggest to include assays with modified PCR methods and to creatively vary PCR conditions if amplification fails or if it leads to 'peculiar' results [see also [54]].

We show that the practical amplification issues of 18S are conquerable, whereas the future of mollusk class level phylogeny appears not to lie in this gene. The incorporation of additional data, such as structural information to identify homologous positions between sequences, is a promising approach to improve alignments and phylogenetic analyses. Within the Mollusca, where the sequence composition is highly variable between clades, both alignment methods and models of evolution used in phylogenetic analyses at date still remain the main bottlenecks in tracing deep phylogeny. Thus, multigene analyses are more effective to resolve such ancient splits, as recently demonstrated [55].

## Methods

### Animals and PCR conditions

All Solenogastres specimens were collected off Bergen, Norway and starved up to three months at 4°C in natural seawater. RNA was extracted from six to twenty animals of each species using Trizol (Invitrogen, Germany) and followed by DNase digestion on NucleoSpin II columns (Macherey-Nagel, Germany). RT was performed for 45 min at 55°C using 100 ng of RNA as template. Either random or the gene specific R1843 primer [56] were applied using Superscript III (Invitrogen, Germany) or BcaBEST™ RNA PCR Kit Ver. 1.1 (TaKaRa Bio Inc., Japan). The GC-rich PCR system (Roche) was used to amplify cDNA adding the supplied GC-rich resolution solution to a final concentration of 0.5 M. Three non overlapping fragments were amplified in all species. The thermal profile for the primers 500F (5'GCGGCGCGACGATCGAAATGAGTCGG3') and 2000R (5'GCCTTATCCCGAGCACGCGCGGGGTTCG3'), annealing at 58°C was setup as time incremental PCR with 1'35" + 5"/cycle at 72°C for 25 times, and a final elongation at 68°C for 7'. The two smaller, neighboring 18S regions were annealed at 53°C [primer F19 [57] and 500R (5'CCGACTCATTTCGATCGTCGCGCCGC3')] or 56°C [primer 2000F (5'CGAACCCCGCGCGTGCTCGG3')/R1843 [56]]. All PCR products were excised from 0.8% TAE agarose gels and isolated using the Agarose-Out kit (EURx, Poland). All large central 18S PCR fragments (500F/2000R) were cloned using the TOPO-TA vector (Invitrogen, Germany) and electrocompetent cells DH10B pulsed at 1800 V. Recombinant plasmids from over night grown liquid cultures (1.5 ml) were isolated using the GeneMatrix Miniprep purification kit (EURx, Poland). Sequencing was performed using a M13 primer, gene specific primer or Wiar900F (CCGCGGCCGCCTCG) and R427 [58]. Cycle sequencing was done applying the Taq Dye Terminator system (Big Dye 3.1, Applied Biosystems) including 10% DMSO in each reaction. All sequences were submitted to Genbank: *Wirenia argentea *FJ649599, *Simrothiella margaritacea *FJ649600 and *Micromenia fodiens *FJ649601.

### Sequence conformation (figures 1 and 2)

Melting curves of double stranded DNA products were analyzed with Stitch Profiles [27]. We determined the critical temperature to melt half of the DNA (helicity = 0.5) in *Wirenia argentea *and than applied 90.7° using the Blossey and Carlon [59] parameter set. The profiles were aligned to the public available sequences for *Epimenia babai *(AY212107, AY212106), *Epimenia *sp. (AY377657) and *Helicoramenia *sp. (AY145377, AY212108). Secondary structures were calculated at 72°C and 65°C with MFold [60] at the Institut Pasteur webserver http://bioweb2.pasteur.fr.

### Sequence composition (table 1)

18S data (>1.6 kb) from 834 mollusks and 40 non-mollusk outgroup species including all sequence data available for Sipunculida (11 species) and Kamptozoa (5 species) were collected from GenBank and aligned using Mafft version 6.7 [61,62] in the E-INS-i mode. GC content, gene length, base composition distance and the disparity index were calculated from this alignment using Mega version 4.0 [29]. The secondary structure from *Mimachlamys varia *L49051 was extracted from the European Ribosomal Database and used to fit a consensus secondary structure on the alignment using RNA-Salsa [63] in combination with the Alicut perl script http://zfmk.de/web/Forschung/Abteilungen/AG_Wgele/Software/Utilities/index.en.html. Ambiguously aligned hypervariable loop regions within the 870 taxa alignment were identified by eye and coded as binary absence (gap) and present (any base) data [64] before fed into RAxML version 7.2.2 [65]. The S16 model was used for paired sites, the GTR + Γ for non paired sites and the two states BIN model for ambiguously aligned regions. The resulting ML tree was used to infer the branchlengths from each species tip to the most basal node (last common ancestor of *Priapulus caudatus *and all remaining lophotrochozoans) using the tool Branchlength at the HIV database http://www.hiv.lanl.gov/content/sequence/HIV/HIVTools.html.

### Alignment and phylogenetic inferences

The species chosen for phylogenetic inferences aimed to represent the molecular diversity observed in the 870 taxa alignment. If possible, short branching species were preferred. An alignment of 123 species was completed using Mafft (see above) and subsequently trimmed using Aliscore [66]. A guide tree was constructed from this alignment in RAxML 7.2.2 using the GTR + Γ model and a constrain tree for the molluscan classes. The resulting ML tree was used as guide tree in Prank_+F _[67] with two subsequent alignment steps using the flags -once, -realbranches and maxbranches = 0.15 respectively. A consensus secondary structure was provided as described in the previous section. The same alignment procedure was repeated for a second alignment including three Aeolidina (Heterobranchia). The final alignment was edited by eye and trimmed using a python script (available from the authors) in combination with Alicut to discard columns with a threshold of 90% missing data while maintaining a valid secondary structure file. Prank is very conservative in detecting homologous positions and thus this relatively low cut off already provides dense high quality alignments. The trimmed 123 taxa alignment was used to calculate the disparity index and to test the substitution pattern with 1,000 Monte Carlo replicates as implemented in Mega 4.0.

ML trees for all 14 doublet models implemented in RAxML were calculated to select the best fitting substitution model from the 6-, 7-, and 16-state category of models, respectively, applying the AIC as described in Tsagkogeorga et al. [9]. For each of these models we conducted 200 inferences on the original alignment to search for the best ML tree and 1,000 bootstrap replicates enabling the bootstopping option for the frequencies based criterion [68]. The inferences were repeated with RY (G, A = R; C, T = Y) coded loop regions. The wRF distance between the three ML trees was calculated using RAxML 7.2.2.

Phase version 2.0 http://www.bioinf.manchester.ac.uk/resources/phase/ was used for the bayesian inference. The burnin for each setting was determined conducting fully sampled 3,000,000 generation pre-runs (burnin = 0). We adapted the settings from the example control file tol40.mcmc according our dataset in changing the models to GTR + Γ_6 _and S7B + Γ_6 _with the proposal priorities for model 1 to = 7 and model 2 to = 24. All chains sampled 10,000,000 generations, discarding 2,000,000 generations as burnin. File conversion was performed using phase2tracer.pl http://hymenoptera.tamu.edu/rna/download.php. The best model found was optimized using the phase tool Optimizer to generate a model block with fixed substitution rates for a time heterogeneous run. The time-heterogeneous run estimated the base frequencies separately for three independent (GTR + Γ_6_, S7B + Γ_6_) models. Four chains run analogous as described for the mixed model above and additionally a fifth chain sampled 50,000,000 generations, discarding 10,000,000 generations, to determine the consensus tree and Bayesian posterior probabilities. Convergence of likelihood values and parameters was monitored using Tracer [69].

## Authors' contributions

BL and CT set up and supervised this study, AM and NTM conducted PCR experiments and sequencing, AM performed secondary structure calculation, phylogenetic analysis and estimation of substitution rates of sequence data. AM, CT and BL wrote the manuscript. All authors read and approved the final manuscript.

## Supplementary Material

Additional file 1 **Blast result table**. BLASTn results from previously published Solenogastres sequences using strings within regions of strong secondary structure (helices 1-3; see figure 1a). The short fragments of *Epimenia babai *18S lie outside of helix regions and do not contain alien DNA. For the two published helix regions of *Epimenia *sp., the query results in Cnidaria (27 matches within Octocorallia). In all *Helicoradomenia *fragments the 18S of the polychaete *Amphisamytha galapagensis *was found within the six best BLASTn hits (excluding *Helicoradomenia*; mean = 3), while the first hit for Mollusca was on rank 16 (mean = 35). Click here for file

Additional file 2 Likelihood traces from four chains using the heterogeneous model and sampling 10,000,000 generations each. Click here for file
